# Defective mitophagy in aged macrophages promotes mitochondrial DNA cytosolic leakage to activate STING signaling during liver sterile inflammation

**DOI:** 10.1111/acel.13622

**Published:** 2022-05-22

**Authors:** Weizhe Zhong, Zhuqing Rao, Jian Xu, Yu Sun, Haoran Hu, Ping Wang, Yongxiang Xia, Xiongxiong Pan, Weiwei Tang, Ziyi Chen, Haoming Zhou, Xuehao Wang

**Affiliations:** ^1^ Hepatobiliary/Liver Transplantation Center The First Affiliated Hospital of Nanjing Medical University Nanjing China; ^2^ Key Laboratory of Liver Transplantation Chinese Academy of Medical Sciences Nanjing China; ^3^ NHC Key Laboratory of Living Donor Liver Transplantation Nanjing Medical University Nanjing China; ^4^ Department of Anesthesiology The First Affiliated Hospital with Nanjing Medical University Nanjing China

**Keywords:** aging, macrophage, mitochondrial DNA, mitophagy, sterile inflammation, stimulator of interferon genes

## Abstract

Macrophage‐stimulator of interferon genes (STING) signaling mediated sterile inflammation has been implicated in various age‐related diseases. However, whether and how macrophage mitochondrial DNA (mtDNA) regulates STING signaling in aged macrophages remains largely unknown. We found that hypoxia‐reoxygenation (HR) induced STING activation in macrophages by triggering the release of macrophage mtDNA into the cytosol. Aging promoted the cytosolic leakage of macrophage mtDNA and enhanced STING activation, which was abrogated upon mtDNA depletion or cyclic GMP‐AMP Synthase (cGAS) inhibition. Aged macrophages exhibited increased mitochondrial injury with impaired mitophagy. Mechanistically, a decline in the PTEN‐induced kinase 1 (PINK1)/Parkin‐mediated polyubiquitination of mitochondria was observed in aged macrophages. *Pink1* overexpression reversed the inhibition of mitochondrial ubiquitination but failed to promote mitolysosome formation in the aged macrophages. Meanwhile, aging impaired lysosomal biogenesis and function in macrophages by modulating the mTOR/transcription factor EB (TFEB) signaling pathway, which could be reversed by Torin‐1 treatment. Consequently, *Pink1* overexpression in combination with Torin‐1 treatment restored mitophagic flux and inhibited mtDNA/cGAS/STING activation in aged macrophages. Moreover, besides HR‐induced metabolic stress, other types of oxidative and hepatotoxic stresses inhibited mitophagy and promoted the cytosolic release of mtDNA to activate STING signaling in aged macrophages. STING deficiency protected aged mice against diverse types of sterile inflammatory liver injuries. Our findings suggest that aging impairs mitophagic flux to facilitate the leakage of macrophage mtDNA into the cytosol and promotes STING activation, and thereby provides a novel potential therapeutic target for sterile inflammatory liver injury in aged patients.

## Graphical abstract

Metabolic, oxidative stress and drug toxicity induced stress causes macrophage mitochondrial damage to promote cytosolic mtDNA release and STING activation. Mitophagy serves as an important negative feedback regulatory mechanism to mediate STING activation by degrading damaged mitochondia. Aging impairs mitophagy activation by inhibiting PINK1‐mediated mitochondrial ubiquitination and lysosome biogenesis and function.

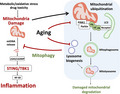

## 1 INTRODUCTION

Sterile inflammation is a form of pathogen‐free inflammation primarily caused by ischemia, taxitic drugs, or mechanical trauma. Emerging evidence indicates that aging contributes to the pathogenesis of various diseases by promoting inflammation (Sato & Yanagita, 2019; Tyrrell & Goldstein, 2021). The cGAS/STING pathway is a vital natural sensor that initiates the host immune response against different pathogens, such as bacteria and viruses (Zhang et al., 2020). Recent studies have revealed the function of cGAS/STING signaling in regulating sterile inflammatory diseases, such as autoinflammatory and autoimmune diseases, and cancer (Ablasser & Chen, 2019). During cellular stress and tissue damage, mtDNA serves as an important danger‐associated molecule that leads to cGAS/STING and inflammation activation (Riley & Tait, 2020). Therapies targeting the cGAS/STING pathway have been suggested as treatment alternatives for various inflammatory diseases (Decout et al., 2021).

Macrophage‐STING signaling mediated sterile inflammation has been implicated in several liver diseases, such as nonalcoholic fatty liver disease (NAFLD), alcoholic liver disease (ALD), and liver cancer (Xu et al., 2021). Damage‐associated molecular patterns (DAMPs) from injured liver parenchymal cells induce the activation of macrophages via various types of pattern recognition receptors (PPRs), leading to an inflammatory response and liver injury. In a previous study, we found that STING activation in macrophages contributed to excessive sterile inflammation and aggravated ischemia and reperfusion (IR) injury in aged livers (Zhong et al., 2020). However, the mechanism underlying the aging dependent increase in STING activation in macrophages remains largely unknown.

Mitophagy leads to the elimination of dysfunctional or superfluous mitochondria via cellular autophagy and serves as the primary mechanism for mitochondrial quality control (Palikaras et al., 2018). Many studies have demonstrated that mtDNA from parenchymal cells can serve as a cGAS ligand and activate cGAS/STING signaling (Zhang et al., 2020); however, it is not well understood if endogenous mtDNA from macrophages can activate STING signaling in macrophages. Given that autophagy has been reported to be defective in aged macrophages (Stahl et al., 2018), we hypothesized that impaired mitophagy in aged macrophages may cause mitochondrial injury and the subsequent cytosolic release of mtDNA, leading to STING activation and induction of proinflammatory responses.

In the present study, we found that aging impaired mitophagy activation in macrophages. This deficiency in the activation of mitophagy leads to the cytosolic release of mtDNA and further promoted STING activation in macrophages. Defects in both, PINK1/Parkin‐mediated polyubiquitination of mitochondria, and mTOR/TFEB‐mediated lysosomal biogenesis and function contributed to the impaired mitophagic flux in aged macrophages. STING deficiency attenuated various types of sterile inflammatory liver injuries in aged mice. The findings from this study might advance our understanding of the interplay between mitophagy dysfunction and STING activation in aged macrophages during sterile inflammatory liver injury.

## 2 RESULTS

### 2.1 Increased leakage of mtDNA into the cytosol promoted STING activation in aged macrophages

**FIGURE 1:**
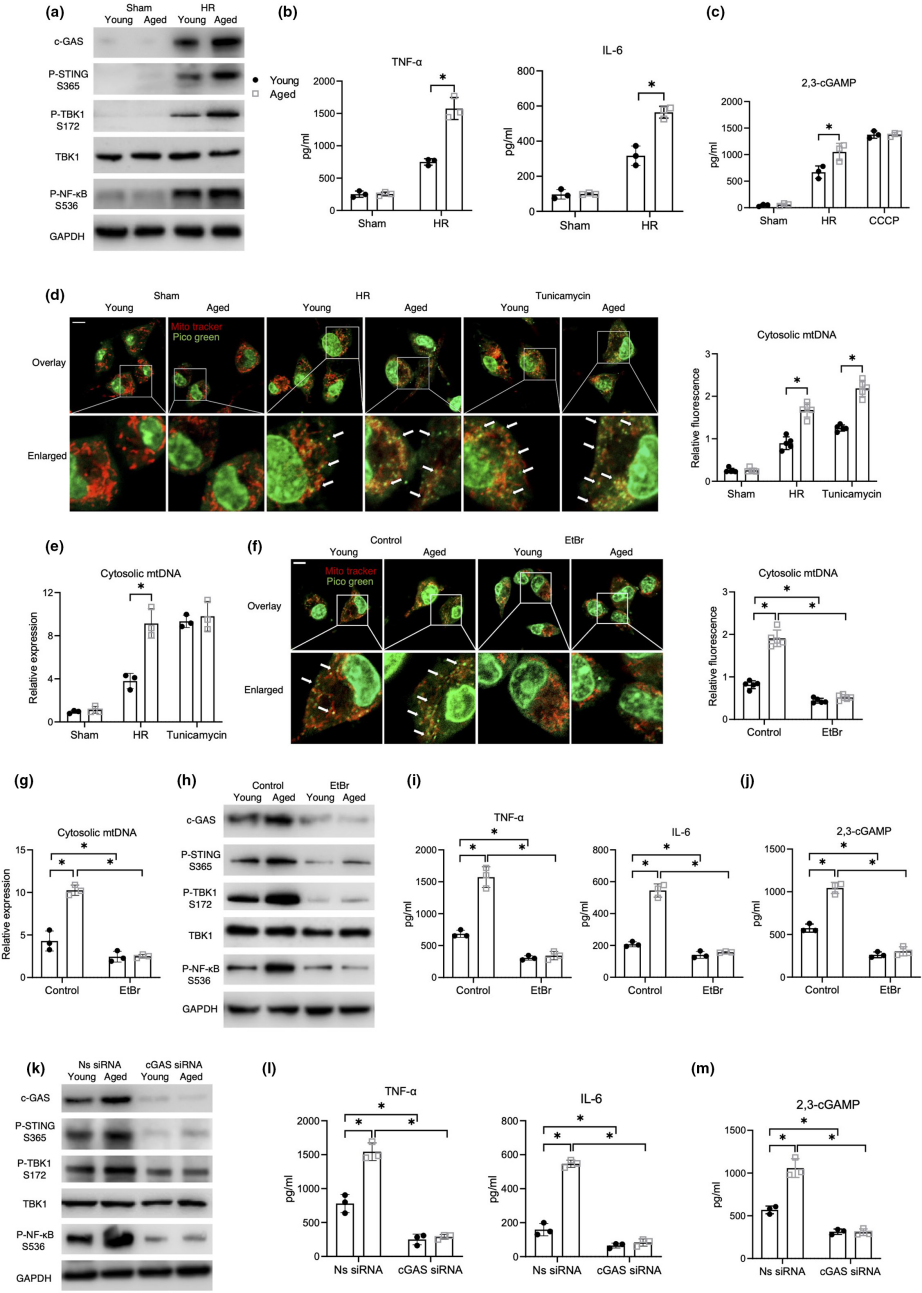
Increased leakage of mtDNA into the cytosol promoted STING activation in aged macrophages. (a, b) Young and aged BMDMs were subjected to HR as described in the section of methods. (a) Immunoblotting of cGAS, P‐STING S365, P‐TBK1 S172, TBK1, P‐NF‐κB S536, and GAPDH. (b) ELISA analysis of TNF‐α and IL‐6 in medium supernatant (*n* = 3). (c) ELISA analysis of intracellular 2,3‐cGAMP (*n* = 3). (d) Confocal microscopy images of BMDMs stained with MitoTracker Red and PicoGreen (scale bar, 10 μm). Relative fluorescence intensity of cytosolic mtDNA was plotted (*n* = 5). Tunicamycin served as a positive control (4 μg/ml). (e) qRT‐PCR assessment for cytosolic mtDNA (*n* = 3). (f–i) The BMDMs were treated with EtBr (400ng/ml). (f) Immunofluorescent imaging (scale bar, 10 μm) and (g) qRT‐PCR analysis of cytosolic mtDNA confirming mtDNA depletion by EtBr (*n* = 3). (h) Immunoblotting of cGAS, P‐STING S365, P‐TBK1 S172, TBK1, P‐NF‐κB S536, and GAPDH. (i) ELISA analysis of TNF‐α and IL‐6 in medium supernatant (*n* = 3). (j) ELISA analysis of intracellular 2,3‐cGAMP (*n* = 3). (k–m) The BMDMs were pre‐transfected with cGAS siRNA or Ns siRNA for 36 h before HR as described in the section of methods. (k) Immunoblotting of cGAS, P‐STING S365, P‐TBK1 S172, TBK1, P‐NF‐κB S536, and GAPDH. (l) ELISA analysis of TNF‐α and IL‐6 in medium supernatant (*n* = 3). (m) ELISA analysis of intracellular 2,3‐cGAMP (*n* = 3). All these immunoblots have been repeated for 3 times. Data are presented as mean ± SD. *p < 0.05

We previously found that enhanced STING activation in aged macrophages contributes to increased ischemic liver injury. Numerous studies have reported on STING activation in macrophages by the invading pathogens or mtDNA released from parenchymal cells (Zhang et al., 2020). A recent study demonstrated that cytosolic mtDNA was able to induce STING activation in fibroblasts (Rai et al., 2021). However, whether mtDNA from macrophages can activate STING signaling is not well described. Therefore, we used the HR model of bone marrow derived macrophages (BMDMs) to investigate the role of macrophage mtDNA in STING activation. HR stress induced the activation of cGAS/STING/TANK‐binding kinase 1 (TBK1)/NF‐κB signaling and the secretion of proinflammatory cytokines, such as tumor necrosis factor‐α (TNF‐α) and interleukin‐6 (IL‐6), as well as intracellular 2,3‐cGAMP levels in macrophages. This effect was further enhanced in aged macrophages (Figure 1a–c). Moreover, the cytosolic leakage and accumulation of mtDNA was found in both, young and aged macrophages after HR stress or tunicamycin treatment, as shown by immunofluorescent staining (Figure 1d) and polymerase chain reaction (PCR) analysis of cytosolic mtDNA (Figure 1e). Cytosolic fractions were isolated by the Mitochondria/Cytosol Fractionation Kit. The purity was confirmed by Western blot analysis of TIM23 (Figure S1a) and qPCR analysis of nuclear tert DNA expression (Figure S1b). The results showed that the levels of remaining mitochondrial contamination were low and did not differ between groups. To determine the role of mtDNA in activating STING signaling in macrophages, we depleted mtDNA from the macrophages using ethidium bromide (EtBr). mtDNA depletion, as confirmed by immunofluorescent staining and PCR (Figure 1f,g), abolished the over activation of GAS/STING/TBK1/NF‐κB signaling in aged mice (Figure 1h–j). Since cGAS senses mtDNA and subsequently activates STING, we further examined whether blocking cGAS could inhibit the macrophage mtDNA induced STING activation. Indeed, we observed that knocking down cGAS inhibited STING signaling and cytokine expression in macrophages after HR and abrogated the differences in STING signaling and cytokine expression between young and aged macrophages (Figure 1k–m). Together, these findings suggested that aging enhanced STING activation in macrophages by promoting the cytosolic leakage of mtDNA.

Several studies have reported that the number of mitochondria decreases with age in liver cells of mice (Herbener, 1976), rats (Stocco & Hutson, 1978), and humans (Yen et al., 1989). Consistently, in the present study, reduced mitochondrial fluorescence intensity were observed in aged macrophages (Figure S1c). Decline in mitochondrial biogenesis of aged cells was found to be responsible for decreased mitochondrial quantity (Huang et al., 2019). We compared the mRNA levels of mitochondrial biogenesis‐related genes in young and aged macrophages and found that peroxisome proliferator‐activated receptor‐gamma coactivator (PGC)‐1a and PGC‐1b mRNA levels were decreased in aged macrophages (Figure S1d), indicating a potential role of aging in reducing mitochondrial biogenesis and mitochondrial number.

### 2.2 Aging promoted mitochondrial injury and impaired mitophagy activation in macrophages

**FIGURE 2:**
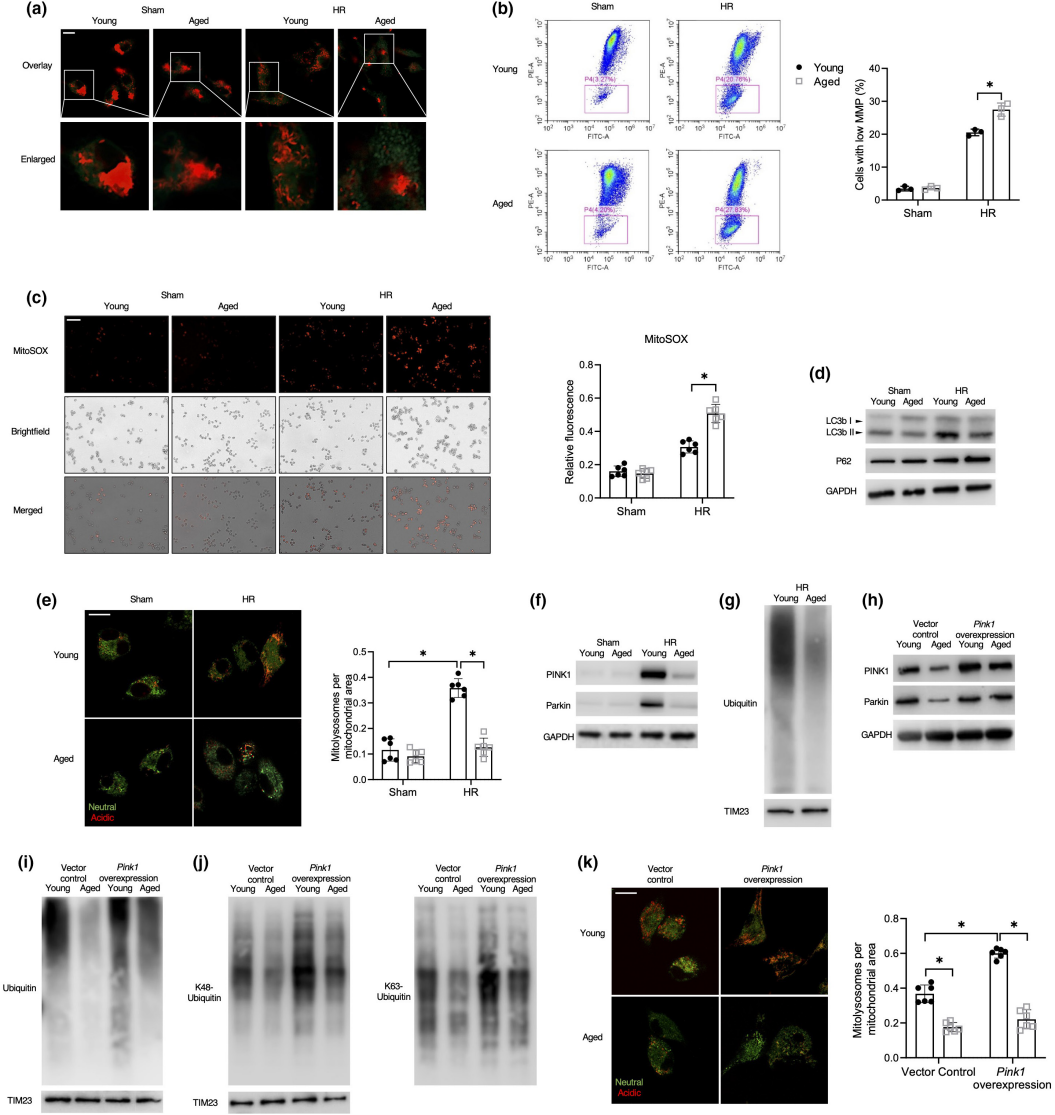
Aging promoted mitochondrial injury and impaired mitophagy activation in macrophages. The BMDMs were subjected to HR as described in the section of methods. (a, b) Detection of MMP by confocal microscopy imaging (a) or FCM (b) (scale bar, 10 μm). The percentage of cells with low MMP was plotted (*n* = 3). J‐aggregates (Red), J‐monomer (Green). (c) ROS activity measurement (scale bar, 250 μm), relative fluorescence was plotted (*n* = 6). (d) Immunoblotting of LC3B, P62, and GAPDH. (e) Confocal microscopy images of mt‐mKeima‐expressing BMDMs (scale bar, 10 μm), analyzed for pixel area in the red channel (Acidic) and normalized to the signal in green channel (Neutral) (*n* = 6). (f) Immunoblotting of PINK1, Parkin, and GAPDH. (g) Immunoblotting of ubiquitin and TIM23 of mitochondria lysis. (h–k) BMDMs were transduced with adenovirus packaged‐plasmid CMV‐*Pink1* (MOI ratio = 500:1) or vector control for 36 h, followed by HR treatment. (h) Immunoblotting of PINK1, Parkin, and GAPDH. (i) Immunoblotting of ubiquitin and TIM23 of mitochondria lysis. (j) Immunoblotting of K48 and K63 linked ubiquitin, and TIM23 of mitochondria lysis. (k) Confocal microscopy images of mt‐mKeima‐expressing BMDMs (scale bar, 10 μm), analyzed for pixel area in the red channel (Acidic) and normalized to the signal in green channel (Neutral) (*n* = 6). All these immunoblots have been repeated for 3 times. Data are presented as mean ± SD. *p < 0.05

Next, we analyzed whether the increased cytosolic release of mtDNA in aged macrophages was caused by exacerbated mitochondrial injury. HR stress induced mitochondrial injury, which was more severe in aged macrophages than young macrophages, as evidenced by the immunofluorescent staining and flow cytometry (FCM) analysis of mitochondrial membrane potential (MMP) (Figure 2a,b). A significant increase in reactive oxygen species (ROS) production was observed in aged macrophages following HR stress (Figure 2c).

Moreover, mitophagy plays a vital role in preserving mitochondrial homeostasis and since it has been reported that mitophagy is impaired in aged livers, we questioned whether impaired mitophagy promoted mitochondrial injury and cytosolic leakage of mtDNA in aged macrophages. HR‐stressed aged macrophages showed decreased levels of the protein LC3B II but increased levels of P62, compared with the young group (Figure 2d). To better assay mitophagy, we transfected BMDMs with mitochondrial mKeima, a fluorescent protein that emits different‐colored signals at an acidic (acidified lysosome) and a neutral (cytosol) pH to enable the measurement of mitochondrial delivery to lysosomes (Katayama et al., 2011). We observed an increase in the fluorescent signals from mitolysosomes in young macrophages post HR, however, which was restricted in aged macrophages (Figure 2e). We further inhibited mitophagy in young macrophages by using 3‐methyladenine (3‐MA) and found enhanced cytosolic mtDNA release and STING inflammation activation after HR (Figure S2b–e). These results indicated that aging impaired the activation of mitophagy and promoted mitochondrial injury in macrophages.

### 2.3 Decline in PINK1/Parkin‐mediated polyubiquitination of mitochondria contributed to mitophagy inhibition in aged macrophages

To elucidate the mechanism via which aging inhibits mitophagy, we analyzed the signaling pathway of mitophagy in BMDMs. We found that the levels of PINK1 and Parkin had significantly increased in HR‐stressed macrophages of young mice, while slightly elevated in the aged group (Figure 2f). PINK1/Parkin signaling contributes to the assembly of ubiquitin chains on the mitochondria and serves as an “eat me” signal for the autophagic machinery. Indeed, we observed decreased mitochondrial ubiquitination in aged macrophages after HR (Figure 2g). *Pink1* overexpression reversed this inhibition of mitochondrial ubiquitination in aged macrophages (Figure 2h,i). Besides, *Pink1* overexpression increased Parkin translocation to mitochondria of young and aged macrophages (Figure S2a). Further analysis showed that K48‐ and K63‐linked ubiquitination of mitochondria suppressed by aging could be reversed by *Pink1* overexpression (Figure 2j). Interestingly, although *Pink1* overexpression promoted mitolysosome formation in young macrophages, no significant effects were observed in aged macrophages (Figure 2k). Together, these findings suggested that inhibition of PINK1/Parkin‐mediated polyubiquitination of mitochondria could only partially explain the aging dependent impairment of mitophagy.

### 2.4 Aging inhibited lysosomal biogenesis and function in macrophages by affecting the mTOR/TFEB signaling pathway

**FIGURE 3:**
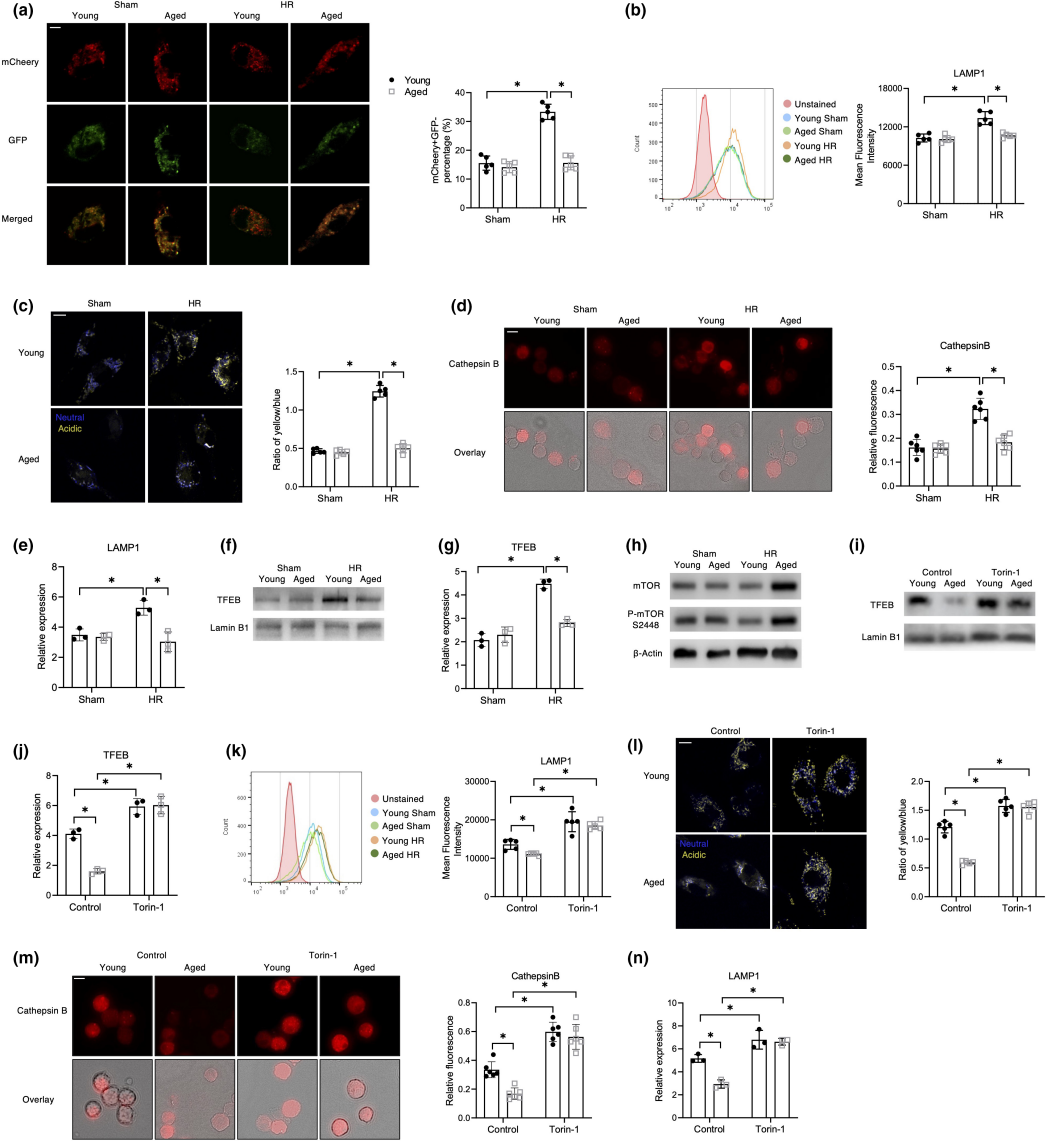
Aging inhibited lysosomal biogenesis and function in macrophages by affecting the mTOR/TFEB signaling pathway. The BMDMs were subjected to HR as described in the section of methods. (a) Confocal microscopy images of mCherry‐GFP‐LC3B‐expressing BMDMs (scale bar, 5 μm). mCheery+GFP‐ percentage was plotted (*n* = 5). (b) FCM analysis of LAMP1 staining. Mean fluorescence intensity was plotted (*n* = 5). (c) Measurement of lysosomal acidification (scale bar, 5 μm). The ratio of yellow/blue fluorescence intensity was plotted (*n* = 5). (d) Measurement of Cathepsin B activity (scale bar, 40 μm). Relative fluorescence was plotted (*n* = 6) (e) qRT‐PCR analysis of LAMP1 (*n* = 3). (f) Immunoblotting of nuclear TFEB and Lamin B1. (g) qRT‐PCR analysis of TFEB (*n* = 3). (h) Immunoblotting of mTOR, P‐mTOR S2448, and β‐Actin. (in). The BMDMs were treated with Torin‐1 (1 μM) for 8 h before HR. (i) Immunoblotting of nuclear TFEB and Lamin B1. (j) qRT‐PCR analysis of TFEB (*n* = 3). (k) FCM analysis of LAMP1 staining. Mean fluorescence intensity was plotted (*n* = 5). (l) Measurement of lysosomal acidification (scale bar, 5 μm). The ratio of yellow/blue fluorescence intensity was plotted (*n* = 5). (m) Measurement of Cathepsin B activity (scale bar, 40 μm), relative fluorescence was plotted (*n* = 6). (n) qRT‐PCR analysis of LAMP1 (*n* = 3). All these immunoblots have been repeated for 3 times. Data are presented as mean ± SD. *p < 0.05

Since *Pink1* overexpression failed to restore the mitophagic flux, we further tested whether autophagosome‐lysosome fusion was also impaired in aged macrophages. To monitor the fusion of autophagosomes with lysosomes, BMDMs were transfected with mCheery‐GFP‐LC3B, and then, confocal microscopy was used to measure the number of yellow (autophagosome) and red (autolysosome). Exposure to the acidic environment of the autolysosomes diminished the GFP fluorescence, whereas the mCherry+signal remained stable. Fluorescent signal of puncta changing from yellow to red indicated the fusion of autophagosomes and lysosomes (Nyfeler et al., 2011). Results showed that significant less red (autolysosome) LC3B puncta was observed in aged macrophages post HR (Figure 3a). FCM analysis revealed that there were fewer lysosomes in aged macrophages than in young macrophages (Figure 3b). Aged macrophages also exhibited reduced lysosomal activity, indicated by decreased lysosomal acidification (Figure 3c), and downregulated expressions of protease cathepsin B (Figure 3d) and the functional protein called lysosomal‐associated membrane protein 1 (LAMP1) (Figure 3e).

The underlying molecular pathways of lysosome biogenesis and function that were affected by aging were also analyzed. TFEB, a master regulator for the generation and function of autophagosomes and lysosomes, was significantly inhibited in aged macrophages (Figure 3f,g). An increase in mTOR activation was observed in aged macrophages after HR stress (Figure 3h). Furthermore, Torin‐1, which is an mTOR inhibitor that triggers lysosomal biogenesis, enhanced TFEB activation (Figure 3i,j), increased the number of lysosomes (Figure 3k), lysosomal acidification (Figure 3l), cathepsin B activation, and LAMP1 expression (Figure 3m,n). Together, these results indicated that aging impaired lysosome biogenesis and function by modulating mTOR/TFEB signaling.

### 2.5 Combination of Pink1 overexpression and Torin‐1 treatment restores mitophagic flux to inhibit cGAS/STING activation in aged macrophages

**FIGURE 4:**
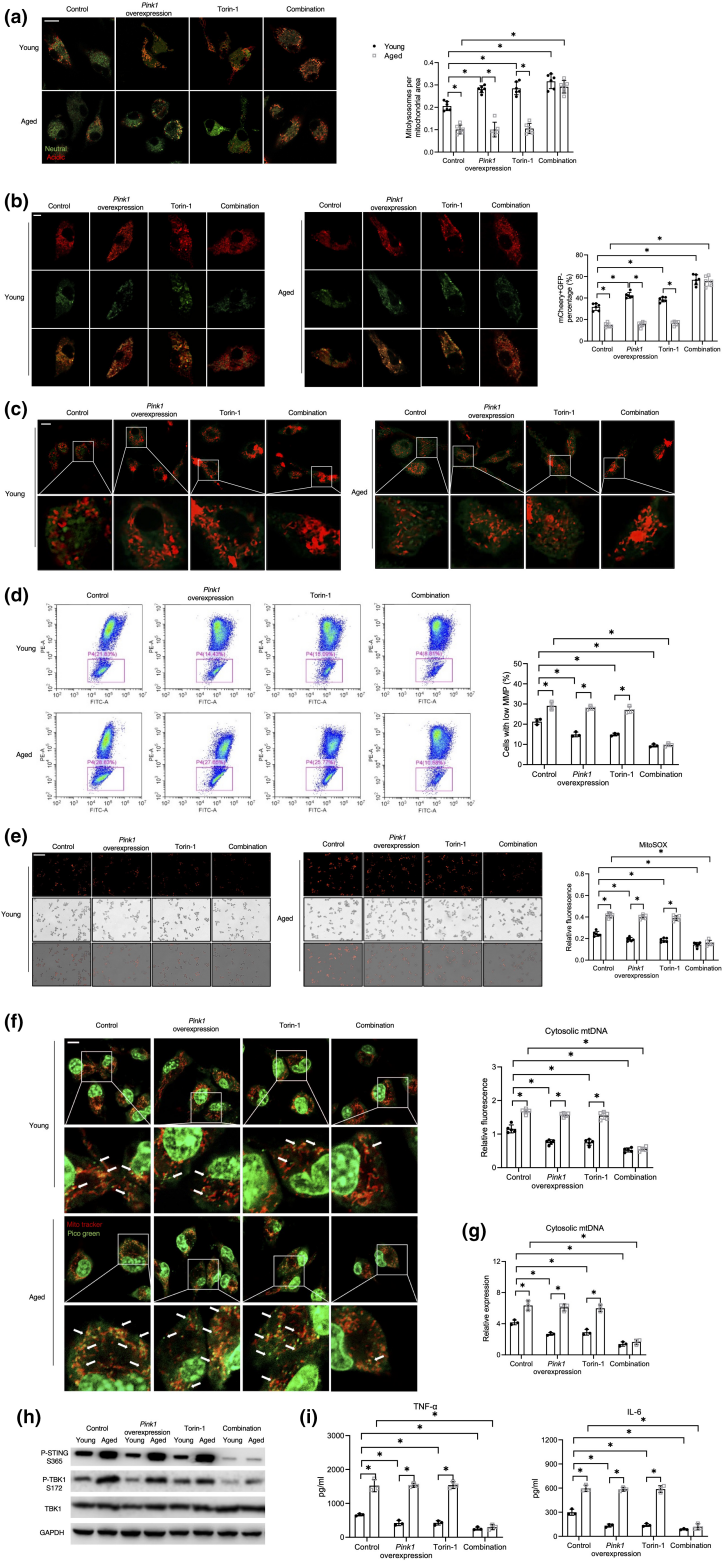
Combination of *Pink1* overexpression and Torin‐1 treatment restored mitophagic flux to inhibit cGAS/STING activation in aged macrophages. BMDMs were transduced with adenovirus packaged‐plasmid CMV‐*Pink1* (MOI ratio = 500:1) for 36 h, followed by HR treatment. Torin‐1 (1 μM) was treated for 8 h followed by HR treatment. (a) Confocal microscopy images of mt‐mKeima‐expressing BMDMs (scale bar, 10 μm), analyzed for pixel area in the red channel (Acidic) and normalized to the signal in green channel (Neutral) (*n* = 6). (b) Confocal microscopy images of mCherry‐GFP‐LC3B‐expressing BMDMs (scale bar, 5 μm). mCheery+GFP‐ percentage was plotted (*n* = 5). (c, d) Detection of MMP by confocal microscopy imaging (scale bar, 10 μm) or FCM. The percentage of cells with low MMP was plotted (*n* = 3). J‐aggregates (Red), J‐monomer (Green). (e) ROS activity measurement (scale bar, 250 μm), relative fluorescence was plotted (*n* = 6). (f) Confocal microscopy images of BMDMs stained with MitoTracker Red and PicoGreen (scale bar, 10 μm). Relative fluorescence intensity of cytosolic mtDNA was plotted (*n* = 5). (g) qRT‐PCR analysis of cytosolic mtDNA (*n* = 3). (h) Immunoblotting of P‐STING S365, P‐TBK1 S172, TBK1, and GAPDH. (i) ELISA analysis of TNF‐α and IL‐6 (*n* = 3). All these immunoblots have been repeated for 3 times. Data are presented as mean ± SD. *p < 0.05

Since both, mitochondrial ubiquitination, and lysosomal biogenesis and function contributed to the impairment of mitophagic flux in aged macrophages, we further evaluate the synergistic effects on macrophage mitophagy of combination of *Pink1* overexpression and Torin‐1 treatment. Indeed, *Pink1* overexpression in combination with Torin‐1 treatment, but not *Pink1* overexpression or Torin‐1 treatment alone, effectively reversed the aging dependent decrease in mitophagic flux in aged macrophages (Figure 4a,b).

Functionally, restoring mitophagy decreased mitochondrial injury in aged macrophages after HR stress, as shown by the analysis of MMP (Figure 4c,d) and ROS (Figure 4e). Moreover, mitophagy restoration abrogated the aging dependent cytosolic leakage of mtDNA in macrophages (Figure 4f,g), subsequent activation of STING signaling (Figure 4h), and inflammatory response (Figure 4i). These findings suggested that aging promoted macrophage mtDNA mediated activation of cGAS/STING by inhibiting mitophagy. Both, PINK1/Parkin‐mediated mitochondrial ubiquitination and mTOR/TFEB‐mediated lysosome biogenesis and function played an indispensable role in this aging dependent inhibition of mitophagy.

### 2.6 Mitophagy mediated mtDNA/cGAS/STING signaling served as an extensive regulatory mechanism in different sterile inflammatory responses

**FIGURE 5:**
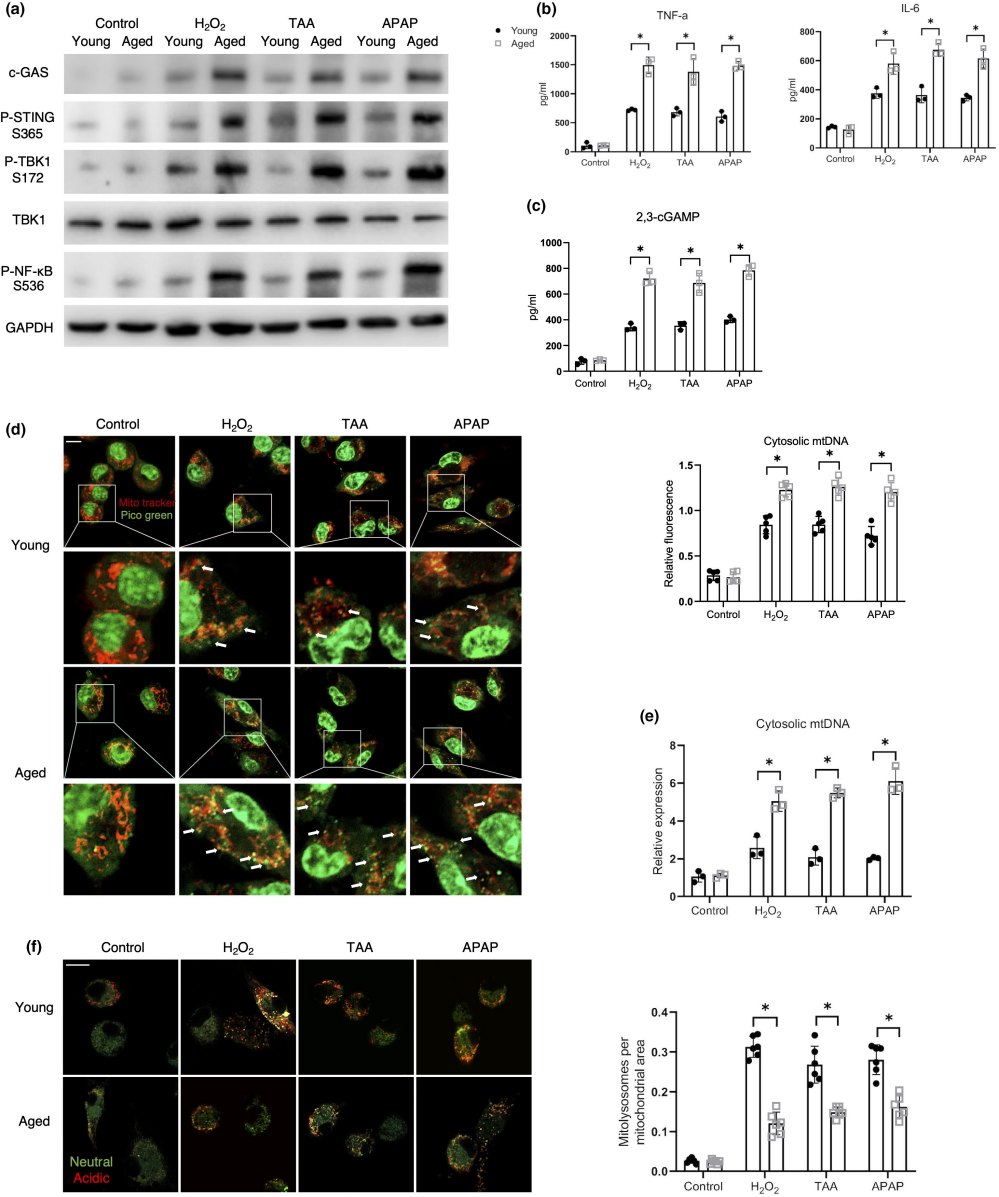
Mitophagy mediated mtDNA/cGAS/STING signaling served as an extensive regulatory mechanism in different sterile inflammatory responses. BMDMs were, respectively, treated with H_2_O_2_ (200 μM, 12 h), TAA (70 μM, 6 h) or APAP (5 mM, 6 h) before HR treatment. (a) Immunoblotting of cGAS, P‐STING S365, P‐TBK1 S172, TBK1, P‐NF‐κB S536, and GAPDH. (b) ELISA analysis of TNF‐α and IL‐6 (*n* = 3). (c) ELISA analysis of intracellular 2,3‐cGAMP (*n* = 3). (d) Confocal microscopy images of BMDMs stained with MitoTracker Red and PicoGreen (scale bar, 10 μm). Relative fluorescence intensity of cytosolic mtDNA was plotted (*n* = 5). (e) qRT‐PCR analysis of cytosolic mtDNA (*n* = 3). (f) Confocal microscopy images of mt‐mKeima‐expressing BMDMs (scale bar, 10 μm) analyzed for pixel area in the red channel (Acidic) and normalized to the signal in green channel (Neutral) (*n* = 6). All these immunoblots have been repeated for 3 times. Data are presented as mean ± SD. *p < 0.05

To determine the function of the mitophagy regulated mtDNA/cGAS/STING signaling in sterile inflammatory stress, other than HR, we treated aged macrophages with oxidative stress (H_2_O_2_) and toxic drugs (thioacetamide [TAA] and acetaminophen [APAP]). Interestingly, akin to our findings in macrophages with HR stress, these stimuli induced STING/NF‐κB activation in macrophages, which was further enhanced in aged macrophages (Figure 5a–c). Aged macrophages also showed increased accumulation of cytosolic mtDNA (Figure 5d,e) and reduced mitophagy activation (Figure 5f) in response to these stimuli.

These results suggested that enhanced mtDNA/cGAS/STING signaling in aged macrophages due to impaired mitophagy contributed to diverse sterile inflammatory responses.

### 2.7 STING deficiency attenuated diverse types of sterile inflammatory liver injury in aged mice

**FIGURE 6:**
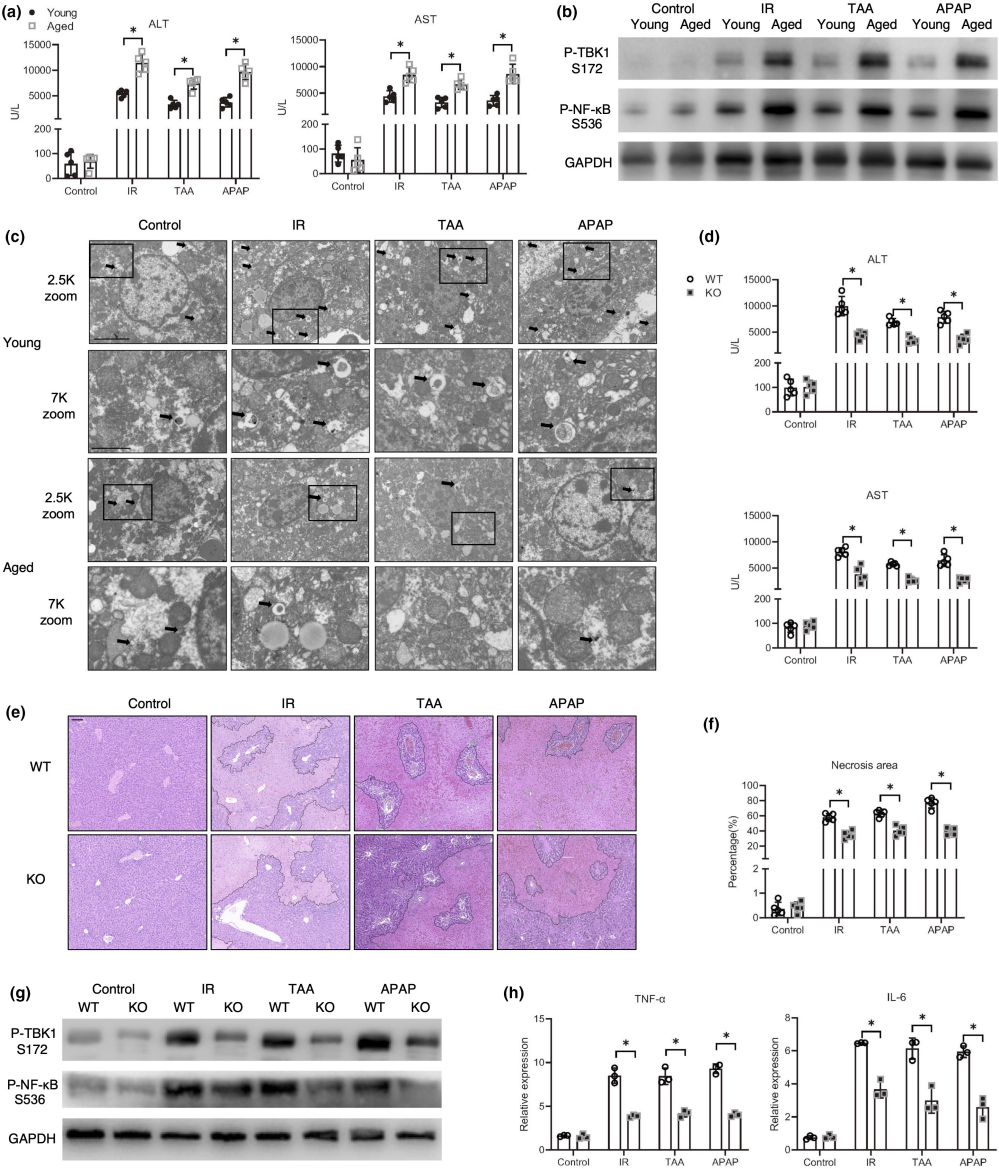
STING deficiency attenuated diverse types of sterile inflammatory liver injury in aged mice. (a–c) Young and aged WT mice were subjected to IR, TAA, or APAP treatment as described in the section of methods. (a) Serum ALT, AST of mice (*n* = 5). (b) Immunoblotting of P‐TBK1 S172, P‐NF‐κB S536, and GAPDH. (c) Transmission electron microscope images of livers. 2.5K (scare bar, 5 μm)/7K (scare bar, 2 μm) X magnification. Mitolysosomes marked by black arrowheads. (d–h) Aged WT and STING KO mice were subjected to IR, TAA, or APAP treatment as described in the section of methods. (d) Serum ALT, AST of mice (*n* = 5). (e, f) Representative HE staining of liver section (scare bar, 100 μm) and the necrosis percentage was plotted. (g) Immunoblotting of P‐TBK1 S172, P‐NF‐κB S536, and GAPDH. (h) qRT‐PCR analysis of TNF‐α and IL‐6 (*n* = 3). All these immunoblots have been repeated for 3 times. Data are presented as mean ± SD. *p < 0.05

To investigate the in vivo role of aging in regulating macrophage mitophagy and STING signaling, diverse types of sterile inflammatory liver injuries were induced in young and aged mice by IR, TAA, or APAP treatment. Aged mice exhibited significantly aggravated liver injury (Figure 6a). Moreover, enhanced STING/NF‐κB activation (Figure 6b) and inhibited mitophagy (Figure 6c) were detected in aged livers after injury.

Finally, we determined the functional significance of macrophage STING signaling in promoting sterile inflammatory liver injury in aged mice. Sterile inflammatory liver injury was induced in WT and STING knockout (KO) mice via IR, TAA, or APAP treatment. In each of the liver injury models, STING deficiency significantly attenuated liver injury in aged mice, as evidenced by the low levels of serum alanine aminotransferase (ALT) and aspartate aminotransferase (AST) (Figure 6d) and better‐preserved liver architectures (Figure 6e,f). Furthermore, STING depletion also abrogated the aging dependent increase in the intrahepatic proinflammatory response, as shown by the analysis for STING/TBK1 signaling activation and TNF‐α and IL‐6 genes (Figure 6g,h). These results suggested that STING deficiency protected aged mice against different types of sterile inflammatory liver injuries.

## 3 DISCUSSION

It has been reported that mtDNA released from parenchymal cells can activate the STING pathway in macrophages during several disease models. In contrast, little is known about whether the release and accumulation of cytosolic mtDNA by stressed macrophages themselves can serve as an intrinsic signal for STING activation in macrophages. In the present study, we found that aging enhanced STING activation in macrophages by promoting the cytosolic leakage of mtDNA during different types of sterile inflammatory responses. Mechanistically, impaired mitophagic flux contributed to an increase in the cytosolic release of mtDNA in aged macrophages. Both PINK1/Parkin‐mediated mitochondrial ubiquitination and mTOR/TFEB‐mediated lysosome biogenesis and function played an indispensable role in the inhibition of mitophagy by aging. STING deficiency protected aged mice against different types of sterile inflammatory liver injuries. Thus, our findings revealed a novel mechanism for the activation of STING signaling in aged macrophages by the macrophage mtDNA that has leaked into the cytosol. Importantly, the mitochondria damage‐induced mitophagy activation serves as an intrinsic regulatory loop to suppress excessive STING activation by clearing damaged mitochondria.

The cGAS/STING signaling pathway serves as a crucial mechanism for detecting pathogenic DNA and triggering an innate immune response during microbial infections (Hopfner & Hornung, 2020). Recently, increasing evidence has indicated that DNA sensors can also be activated by endogenous DNA, such as mtDNA released from the mitochondria. STING signaling in macrophages has been implicated in anti‐tumor immunity, cellular senescence, and autoimmune and inflammatory diseases (Motwani et al., 2019). Endogenous mtDNA released from injured liver parenchymal cells induces STING activation in macrophages, which in turn augments inflammatory liver damage (Xu et al., 2021). During liver IR injury, ROS cause oxidative damage to the mitochondria, leading to the release of cytosolic mtDNA from injured hepatocytes. Repressing STING signaling protects the liver against IR injury (Shen et al., 2020). In contrast, a recent study revealed that cGAS protects the liver from IR injury in a STING‐independent manner (Lei et al., 2018). Due to the limited number of studies, it is still unclear how the cGAS/STING pathway regulates the interplay between hepatocytes and macrophages during sterile inflammatory injury in the liver.

The molecular mechanisms underlying cGAS/STING activation have been well established (Hopfner & Hornung, 2020; Zhang et al., 2020). However, most of these studies focus on the activation of STING signaling in macrophages by the mtDNA released from other parenchymal cells. Moreover, it is not well understood if the leakage of mtDNA into the cytosol, during stress, can activate STING signaling in macrophages. A previous study reported that there was a significant increase in the levels of mtDNA in macrophages after LPS treatment (Ning et al., 2020). In the present study, we observed increased accumulation of mtDNA in the cytosol of stressed macrophages. Next, we investigated and confirmed that macrophage‐derived mtDNA were able to activate STING signaling in these cells. Consistent with our findings, a recent study revealed that cytosolic mtDNA induced cGAS‐STING activation in fibroblasts (Rai et al., 2021).

Mitochondria play a key role in regulating energy generation; oxidative response; cell death, proliferation, and differentiation; and inflammation (Tang et al., 2021). Strategies targeting dysfunctional mitochondrial have shown protective effects against both, chronic and acute liver diseases (Han et al., 2013; Sunny et al., 2017). Dysregulated mitochondrial homeostasis has been reported to activate innate immune responses (Moehlman & Youle, 2020).

Mitochondria quantity decreases with age in liver cells of mice (Herbener, 1976), rats (Stocco & Hutson, 1978), and humans (Yen et al., 1989). Consistently, in the present study, reduced mitochondrial fluorescence intensity were observed in aged macrophages. Decline in mitochondrial biogenesis of aged cells was found to be responsible for the decreased mitochondrial quantity (Huang et al., 2019), indicating a potential role of aging in reducing mitochondrial biogenesis and mitochondrial number.

Mitophagy, the selective autophagic elimination of dysfunctional mitochondria, plays a vital role in mitochondrial quality control (Palikaras et al., 2018). A recent study revealed that prolonged injury (LPS stimulation for 12 h and 24 h) induced mitophagy activation through IL‐10 mediated mTOR inhibition (Sun et al., 2016). Another study also found that NF‐κB exerted its anti‐inflammatory activity by inducing p62/SQSTM1‐dependent mitophagic clearance of damaged mitochondria, which represented a macrophage‐intrinsic regulatory loop to maintain homeostasis and favor tissue repair (Kauppila et al., 2017).

Failure in clearing defective mitochondria via mitophagy causes the cytosolic accumulation of mtDNA and induces a type I interferon response (Rai et al., 2021). PINK1 or Parkin deficiency leads to the accumulation of mutated mtDNA and triggers inflammation via STING activation (Sliter et al., 2018). Impaired mitophagy activation in has been implicated in various diseases (Chen et al., 2020). Aged cells are characterized by the accumulation of dysfunctional mitochondrial, reduced respiratory chain efficacy and adenosine triphosphate (ATP) production, and increased ROS production (Sun et al., 2016). Impaired mitochondrial function causes mutations in mtDNA and contributes to the progression of age‐associated disease and promotes aging (Kauppila et al., 2017). Significant impairment of mitophagy activation causes defective degradation of damaged mitochondria, which plays an important role in the pathogenesis of aging and in age‐related diseases (Chen et al., 2020). The mitophagy activation induced by damaged mitochondria serves as an intrinsic regulatory loop to suppress excessive inflammation by clearing damaged mitochondria. Thus, the impaired mitophagy in aged macrophages caused increased cytosolic mtDNA release from damaged mitochondria to enhance STING activation and inflammation.

cGAS/STING signaling mediated sterile inflammation has been implicated in various age‐related diseases (Paul et al., 2021). Defective autophagy and enhanced inflammatory responses contribute to increased liver injury post IR in aged mice (Kan et al., 2018). In a previous study, we found that enhanced STING activation in macrophages contributed to increased liver IR injury in aged mice (Zhong et al., 2020). However, the underlying mechanism by which aging promotes STING activation in macrophages is unclear. Herein, we evaluated and confirmed that aging impaired mitophagy activation in macrophages, leading to increased mitochondrial injury, cytosolic leakage of macrophage mtDNA, and activation of STING signaling. Our study presents a novel mechanism for STING activation in macrophages.

Cells possess several mitophagy mechanisms that are activated in response to different stimuli and under distinct contexts. Both, ubiquitin‐dependent and ubiquitin‐independent pathways have been implicated in the regulation of mitophagy (Khaminets et al., 2016). PINK1/Parkin signaling promotes the assembly of ubiquitin chains on the mitochondria, which serve as an “eat me” signal. Adaptor proteins then recognize the polyubiquitination chains on mitochondrial proteins and initiate autophagosome formation (Harper et al., 2018). Several studies have shown that the expression levels of both PINK1 and Parkin were increased after mitophagy activation which was induced by various types of stimuli (Chen et al., 2019; Duan et al., 2021; Kong et al., 2022; Lin et al., 2019; Xu et al., 2022) A recent study found that hypoxia induced mitophagy activation accompanying with increased expression levels of PINK1 and Parkin, while *Pink1* knockdown efficiently inhibited mitophagy activation in AGS cells (Xu et al., 2022). Moreover, *Pink1* overexpression was able to activate mitophagy, as demonstrated by the increased expressions of autophagy‐related genes including p62 and ATG16L, elevated ratio of LC3II/LC3I, and mitochondrial acidification (Yin et al., 2021).

It was observed that PINK1 expression is decreased and mitophagy is impaired in aged lungs. Furthermore, *Pink1* depletion promoted lung fibrosis in young mice (Bueno et al., 2015). Consistent with this study, we recorded an inhibition in the activation of PINK1/Parkin signaling in aged macrophages, leading to defective ubiquitin marks on injured mitochondria and impaired mitophagic flux. *Pink1* overexpression increased Parkin translocation to mitochondria of young and aged macrophages. Interestingly, *Pink1* overexpression was insufficient to restore mitophagic flux in aged macrophages, which could be explained by the defective autophagosome‐lysosome fusion due to impaired lysosome function. Aging impaired the lysosomal acidification, which could be reversed by Torin‐1 treatment. Consequently, *Pink1* overexpression effectively restored the mitophagic flux in aged macrophages in combination with Torin‐1 treatment.

## 4 EXPERIMENTAL PROCEDURES

### 4.1 Animals

Young (8 weeks) and aged (100 weeks) male C57/BL6 mice were purchased from Ziyuan Laboratory Animal Technology Co., Ltd. STING KO mice and wild type control mice (WT) were generated by GemPharmatech Co., Ltd. The mice were housed under a 12 h light/dark cycle in a standard specific pathogen‐free environment. All animals received humane care, and all animal procedures met the relevant legal and ethical requirements according to the protocols (number NMU08‐092) approved by the Institutional Animal Care and Use Committee of Nanjing Medical University.

### 4.2 Hepatic IR mouse model

A well‐established model of partial hepatic IR injury was established as described previously (Zhong et al., 2020; Zhou et al., 2018). In brief, after successful anesthesia with 2.5% isoflurane, a midline laparotomy was performed. Then, an atraumatic clip was used to interrupt the blood supply to the left and median lobes of the liver for 90 min. Subsequently, the clip was removed to initiate the process of hepatic reperfusion. The mice that underwent the same procedure but without vascular occlusion, served as controls. The mice were sacrificed after 6 h of reperfusion, and the collected samples were harvested for analysis.

### 4.3 Drug‐induced acute liver injury

Acute liver injury was established by a single intraperitoneal injection of either thioacetamide (TAA; Sigma Aldrich) (150 mg/kg of body weight) or acetaminophen (APAP; Sigma Aldrich) (300 mg/kg of body weight). Samples were harvested 24 h after administration.

### 4.4 Serum transaminase measurement

Serum ALT and AST levels were measured using an AU680 clinical chemistry analyzer (Beckman Coulter).

### 4.5 Histopathology

Liver specimens were fixed in 4% paraformaldehyde and embedded in paraffin for hematoxylin and eosin (H&E) staining.

### 4.6 Culture and treatment of bone marrow derived macrophages (BMDMs)

Bone marrow cells were flushed from the femurs and tibias. After filtration with a 70 μm strainer, the cells were cultured in Dulbecco's modified eagle medium (DMEM) supplemented with 10% FBS, 1% P/S, 1% HEPES, and 20 μg/L colony stimulating factor (CSF; PeproTech) for 7 days. The BMDMs were replated and cultured overnight for further experiments.

For the hypoxia and reoxygenation experiments, the BMDMs were seeded at a density of 1*10^6^ cells/ml in serum and glucose‐free deoxygenated DMEM and maintained in a hypoxic environment (1% O_2_, 94% N_2_, and 5% CO_2_) for 24 h. Subsequently, the medium was removed and replaced with fresh DMEM supplemented with 10% FBS, 1% Penicillin‐Streptomycin (P/S; Gibco), and 1% HEPES (Gibco). Thereafter, cells were incubated under normoxic conditions (21% O_2_, 74% air, and 5% CO_2_) to initiate the reoxygenation for 12 h. The cells and supernatants were collected for further analyses. For in vitro experiments, the BMDMs were treated with H_2_O_2_ at 200 µM for 12 h, TAA at 70 µM for 6 h, or APAP at 5 mM for 6 h.

For mtDNA depletion, EtBr (ethidium bromide; 400ng/ml, Sigma Aldrich) was treated for 48 h (12 h betore HR, 36 h during HR). To inhibit cGAS signaling, mature BMDMs were transiently transfected with cGAS siRNA (10 μM; Santa Cruz Biotechnology) or non‐specific siRNA using Lipofectamine 3000 (Thermo Fisher Scientific) for 36 h before HR. For *Pink1* overexpression, mature BMDMs were transfected with the adenovirus coated‐plasmid CMV‐*Pink1* (multiplicity of infection, MOI ratio = 500:1; ObiO Technology Corp., Ltd.) or vector control for 36 h before HR administration. To inhibit mTOR signaling, mature BMDMs were treated with Torin‐1 (1 μM; Invivogen) for 8 h before HR. For mitophagy inhibition, young BMDMs were treated with 3‐MA (5 mM; Invivogen) for 1 h before HR.

### 4.7 Mitophagy/autophagy assessment

To evaluate mitophagy, BMDMs were transduced with adenovirus packaged‐COX8‐mt‐mkeima (MOI ratio = 200:1; ObiO Technology Corp., Ltd) for 24 h, a mitochondrial‐localizing construct that fluoresces differentially under neutral or acidic conditions. An increase in the red signal indicated higher levels of mitophagic flux. Mitophagy levels were measured by pixel area in the red channel (Acidic) and normalized to the signal in green channel (Neutral). The calculations were based on six randomly selected areas.

To visualize the extent of autophagosome delivery to lysosomes, adenovirus packaged‐mCherry‐GFP‐LC3B (MOI ratio = 200:1; HanBio Technology Corp., Ltd) was administered to the BMDMs for 24 h. The fluorescent signal from GFP was quenched inside the lysosome, but there was no significant change in mCherry under acidic conditions. The autophagosomes are shown as yellow puncta (mCherry+GFP+), while the autolysosomes are shown as red puncta (mCherry+GFP‐). An increase in autolysosomes was considered as promotion in the autophagic flux. According to the previous studies (Kondratskyi et al., 2014), autophagy levels were measured by the percentage of the red puncta (mCherry+GFP‐). The calculations were based on five randomly selected areas.

### 4.8 Mitochondrial measurements

For mtDNA staining, the BMDMs were treated with MitoTracker Red (for mitochondrial staining, 200 nM; Yeasen Biotechnology) for 1 h followed by 15 min of incubation in PicoGreen (for dsDNA staining, 500 ng/ml; Yeasen Biotechnology). Then, the cells were imaged on a Zeiss microscopy with a 63× oil‐immersed objective. To subtract nuclear DNA signal, the PicoGreen‐positive stained nuclear area was deducted by the image processing software. According to the previous studies (Kitajima et al., 2019; Shen et al., 2015), the relative intensity of cytosolic mtDNA was represented by the ratio of intensity of green/red channel post nuclei area deduction. Signal intensity at each region of interest was quantified by ImageJ, and the calculations were based on five randomly selected areas. In addition, tunicamycin (4 μg/ml, 3 h) serviced as a positive control for cytosolic mtDNA release (Aarreberg et al., 2019).

Mitochondrial membrane potential was monitored using the JC‐10 assay kit (Yeasen Biotechnology). In brief, lipophilic dye that got concentrated in healthy mitochondria to form red‐fluorescent J‐aggregates, while in damaged mitochondrial to form green‐fluorescent J‐monomer. A decrease in the red signal was indicative of a reduction in the mitochondrial membrane potential, as measured by cytometry and immunofluorescence. For MMP measurements, we used the percentage of cell with low MMP to compare the overall levels between different groups.

For mitochondrial ROS measurement, MitoSox red (Yeasen Biotechnology) was used at a concentration of 5 μM for 15 min of incubation.

### 4.9 Lysosome measurements

Lysosomal acidification was measured by lysosensor (1 µM for 30 min, Yeasen Biotechnology), a pH‐dependent fluorescent probe, which produced blue fluorescence in neutral environment, but emitted yellow fluorescence in acidic environment. Lysosomal acidification levels were measured by the ratio of yellow/blue fluorescence intensity. The calculations were based on five randomly selected areas.

To quantify lysosomes, BMDMs were treated with the BV421‐LAMP1 antibody (2 μl/10^6^ cells, Biolegend) for 30 min. After staining, the cells were analyzed via flow cytometry.

The activity of Cathepsin B was measured using magic red (Immunochemistry Technologies) according to the manufacturer's instructions.

### 4.10 Quantitative real time polymerase chain reaction (qRT‐PCR)

Total RNA was isolated from cells and tissues using an RNA‐quick purification kit and reverse transcribed into complementary DNA using an PrimeScript RT reagent kit with gDNA Eraser (TaKaRa). qRT‐PCR was performed using a StepOnePlus Real‐Time PCR system (Thermo Fisher Scientific). The final reaction volume was 20 μl, containing 1× TB Green Premix (TaKaRa), complementary DNA, and each primer at 0.125 μM. The amplification conditions were as follows: 50°C for 2 min, 95°C for 10 min, followed by 40 cycles of 95°C for 15 s, and 60°C for 1 min. The 2^−ΔΔCt^method was used to analyze fold changes in gene expression after normalization with GAPDH.

For relative quantification of the cytosolic mtDNA, the Mitochondria/Cytosol Fractionation Kit (Biovision) were used to isolate cytosolic fractions. DNA from the whole cell and cytosolic fractions was isolated using the Dneasy Blood and Tissue kit (Qiagen). qRT‐PCR was performed on both whole cell extracts and cytosolic fractions using mtDNA primer (mtDNA Dloop, Fwd: 5ʹ‐AATCTACCATCCTCCGTGAAACC‐3ʹ, Rev: 5ʹ‐TCAGTTTAGCTACCCCCAAGTTTAA‐3ʹ). The C_t_ values obtained for mtDNA abundance of whole cell extracts served as normalization controls for the mtDNA values obtained from the cytosolic fractions, which allowing effective standardization among samples. Undetectable nuclear tert DNA expression in the cytosolic fraction indicated no nuclear lysis contamination (Fwd; 5ʹ‐CTAGCTCATGTGTCAAGACCCTCTT‐3ʹ, Rev; 5ʹ‐GCCAGCACGTTTCTCTCGTT‐3ʹ) (Bai et al., 2017).

### 4.11 Western blotting

Tissues and cellular proteins were extracted using ice‐cold radioimmunoprecipitation assay lysis buffer (Cell Signaling Technology), supplemented with protease and phosphatase inhibitors (Cell Signaling Technology). The mitochondria fractions were extracted using commercial isolation kit (Abcam), according to the manufacturer's instructions. The nuclear fractions were isolated using Nuclear and Cytoplasmic Protein Extraction Kit (Yeasen Biotechnology). Proteins were subjected to sodium dodecyl‐sulfate polyacrylamide gel electrophoresis (SDS‐PAGE) and transferred to a polyvinylidene fluoride (PVDF) nitrocellulose membrane. Antibodies against cGAS, Phospho‐STING (P‐STING S365), TBK1, Phospho‐TBK1 Ser172 (P‐TBK1 S172), Phospho‐NF‐κB p65 ser536 (P‐NF‐κB S536), Parkin, Ubiquitin, K48‐Ubiquitin, K63‐Ubiquitin, mTOR, Phospho‐mTOR Ser2448 (P‐mTOR S2448), TFEB, Lamin B1, GAPDH (Cell Signaling Technology), PINK1 (Thermo Fisher Scientific), TIM23 (Abcam), and β‐Actin (ProMab Biotechnology) were used and incubated overnight at 4°C. After 2 h of incubation with the appropriate horseradish peroxidase (HRP)‐conjugated secondary antibody, bands were detected with Immobilon ECL Ultra Western HRP substrate (Vazyme Biotechnology), and images were acquired using a Vilber chemiluminescent imaging system (Vilber). Densitometry analysis was performed using the ImageJ software, to determine changes in protein expression.

### 4.12 Enzyme‐linked immunosorbent assay (ELISA)

The secreted cytokines (TNF‐α and IL‐6) and intracellular 2,3‐cGAMP were measured by ELISA, according to the manufacturer's instructions (Thermo Fisher Scientific).

### 4.13 Transmission electron microscopy (TEM) imaging

Livers were dissected from mice and fixed in 4% paraformaldehyde for 12 h followed by 24 h treatment of dehydration. Tissues were processed and observed by transmission electron microscopy. The images were captured at 2.5K/7K magnification.

According to the previous studies (Chakraborty et al., 2020; Klionsky et al., 2021), a dark stained object resembling mitochondria‐like structure inside an autolysosome was recognized as the mitochondrial degradative compartment, representing the completion of mitophagy. In Figure 6c, we used black arrowheads to point these mitolysosomes out.

### 4.14 Data analysis

Two‐tailed student's *t*‐test was applied for comparisons of two groups and one‐way analysis of variance (ANOVA) for comparisons of >2 groups. For all tests, *p* < 0.05 was considered significant.

## CONFLICT OF INTEREST

The authors disclosed no conflicts no interest.

## AUTHOR CONTRIBUTIONS

WZ, HZ, and XW conceived the project, designed experimental strategies, drafted, and revised the manuscript for publication. WZ and ZR performed the experiments and did data analysis. JX, YS, HH, PW, YX, XP, WT, and ZC did the support work. XW, HZ, WZ, and ZR provided funding support and supervised the study.

## Supporting information

Fig S1

Fig S2

## Data Availability

All data supporting the findings of this study are available within the paper.
