# Supplementary figures and images for: Defective mitophagy in aged macrophages promotes mitochondrial DNA cytosolic leakage to activate STING signaling during liver sterile inflammation
Source: Aging Cell. 2022 May 22;21(6):e13622. doi: 10.1111/acel.13622 (PMC9197407; doi:10.1111/acel.13622)

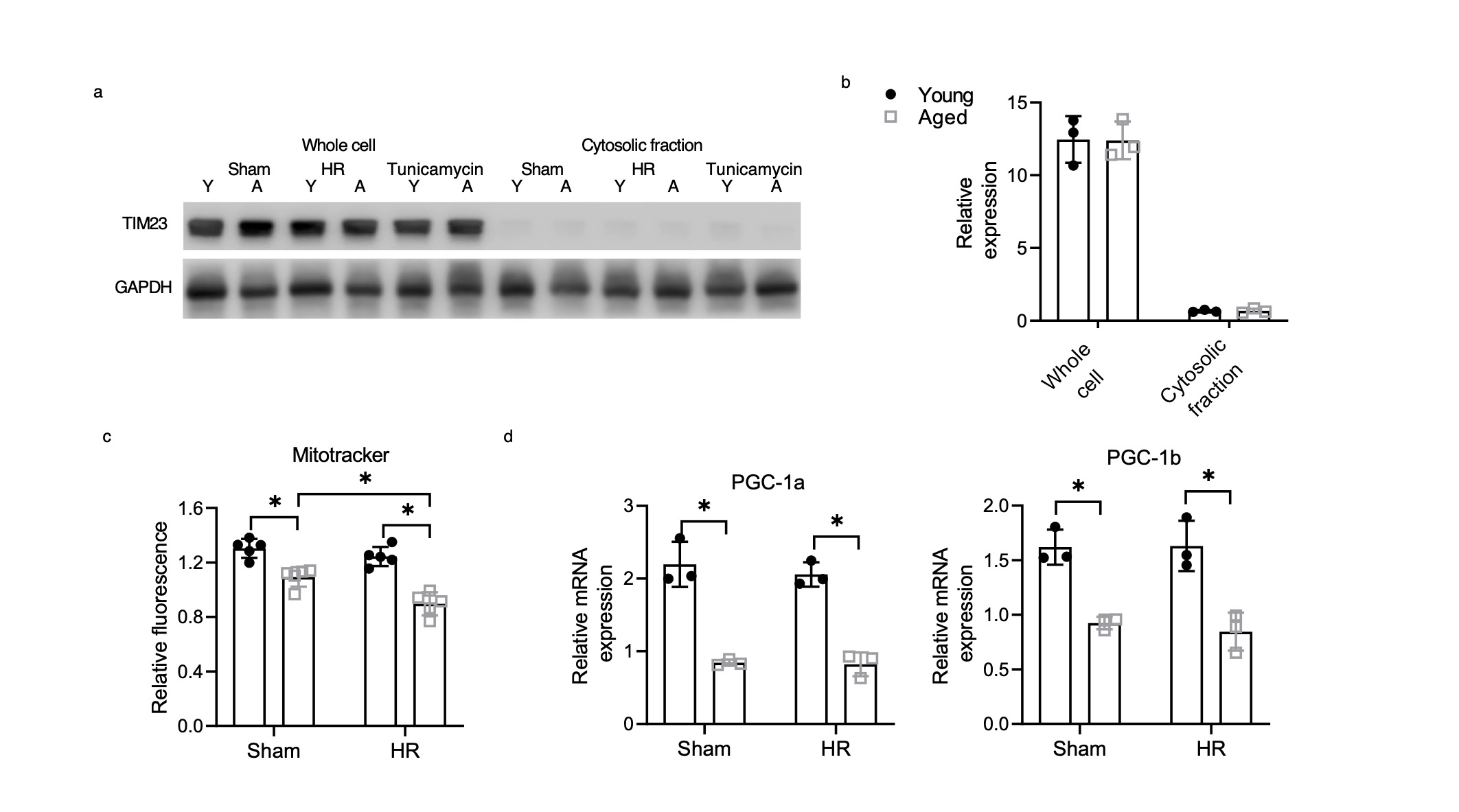

Supplement: Supplementary file 1 — Fig S1 [file ACEL-21-e13622-s002.jpg]

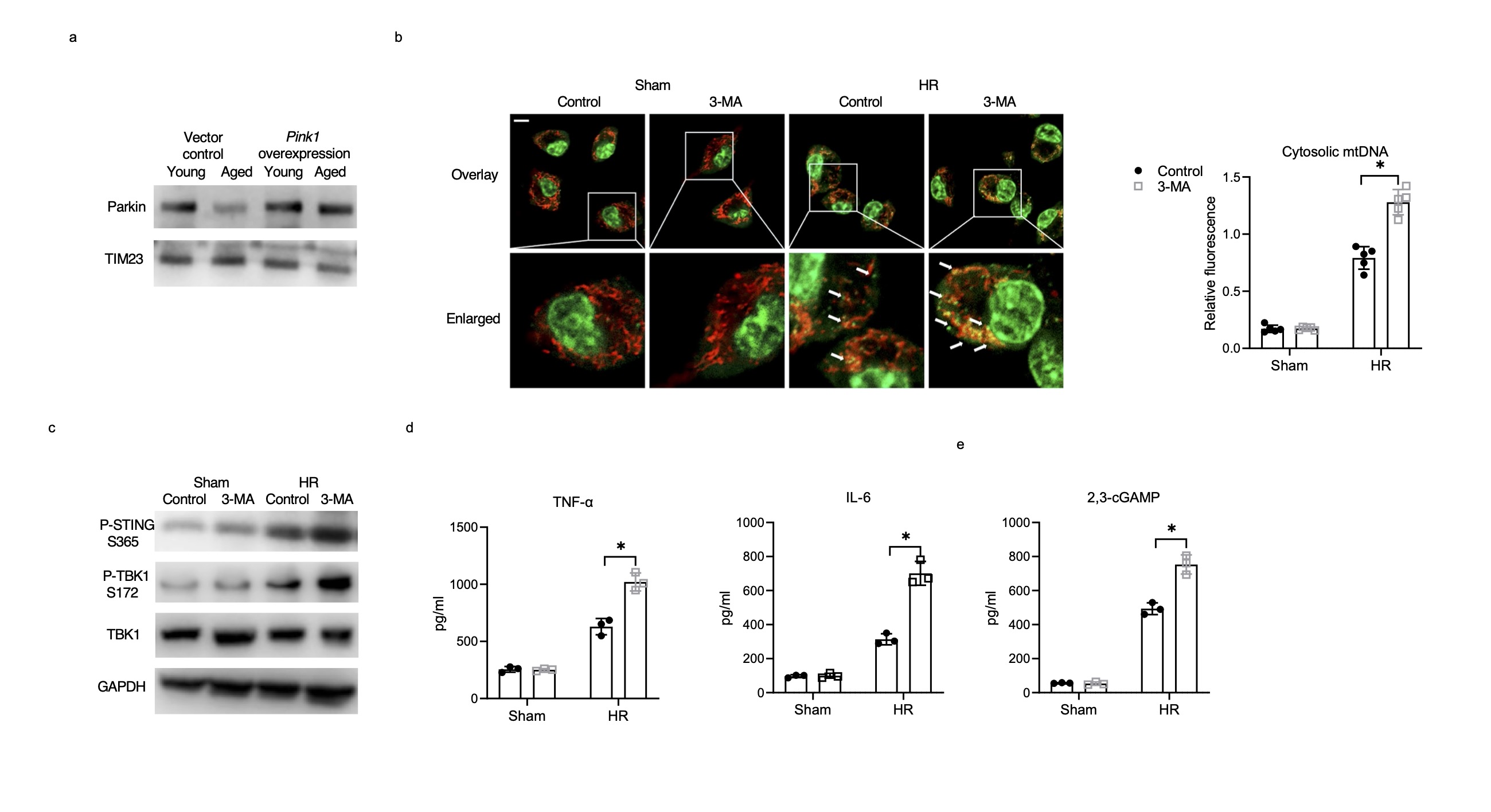

Supplement: Supplementary file 2 — Fig S2 [file ACEL-21-e13622-s001.jpg]
